# Developments in early adolescents’ self-regulation: The importance of teachers’ supportive vs. undermining behavior

**DOI:** 10.3389/fpsyg.2022.1021904

**Published:** 2022-11-23

**Authors:** Marie-Christine Opdenakker

**Affiliations:** Chair Group Education, University of Humanistic Studies, Utrecht, Netherlands

**Keywords:** self-regulation, self-regulated learning, teacher behavior, basic psychological needs, self-determination theory, secondary education, teacher support, adolescence

## Abstract

Research has established that the ability to self-regulate is an important factor in adolescents’ learning, and cognitive and social functioning. Several theories on self-regulation and classroom studies suggest effects of the social learning environment on students’ self-regulation. However, most studies investigating these relations have a cross-sectional correlational design and do not relate to adolescents, resulting in little knowledge about causal directions and adolescents. This study extends existing research by examining effects of a selection of supportive and undermining teacher behavior dimensions on early adolescents’ development of self-regulation (self-regulated learning). The teacher behavior dimensions are based on ideas of the self-determination theory in which a distinction is made between dimensions that support vs. thwart three basic psychological needs (need for autonomy, competence, and relatedness) which are assumed to be important for human growth and (psychological) well-functioning. Supporting autonomy, delivering structure, and being involved with the students are assumed to be important for the fulfillment of students’ basic psychological needs, while exhibiting controlling instructional behavior, having chaos, uncertainty and inconsistency in the classroom, and rejection and neglect of students, are supposed to be a treat. Questionnaires were used for measuring students’ perceptions of their teachers’ behavior and their own self-regulation at several points in time during their first year of secondary education. Participants in the study were 566 students belonging to 20 Mathematics/English grade-7 secondary education classes in The Netherlands. Multilevel analyses point to the importance of all three teacher need-supportive dimensions (with highest effects of structure and involvement) and indicated that teachers’ need-thwarting behavior negatively affected students’ self-regulation. However, when corresponding supportive and thwarting teacher behavior dimensions were included together in the same multilevel model, only the effect of the undermining dimension of controlling teacher behavior remained significant in addition to the corresponding autonomy-support dimension. Findings are in line with existing research and highlight the importance of both teachers’ need-supportive and teachers’ need-thwarting behavior in daily secondary-education classrooms and contribute to deepen our insight in and understanding of factors (related to external regulation by teachers) leading to positive and negative developments of early adolescents’ self-regulation, and, in particular, their self-regulated learning.

## Introduction

Being able to regulate oneself is a very important capacity in life and is often considered as the foundation for lifelong functioning across a wide range of domains since self-regulation plays an important role in relationships, prosocial and moral(ly relevant) behavior, well-being, learning, (academic) achievement, health, and overall success in life (Eisenberg, 2000, 2010; Moffitt et al., 2011; Carlo et al., 2012; Hofmann et al., 2014; Dent and Koenka, 2016; Hampson et al., 2016; Panadero, 2017; Chu et al., 2020). In addition, research has established that it is a predictive factor of resilience (Eisenberg and Spinrad, 2004; Artuch-Garde et al., 2017; de la Fuente-Arias, 2017), and can act as a protective factor for, in particular, youth at risk of social exclusion (Artuch-Garde et al., 2017) and maladaptive social behavior (Gardner et al., 2008). People who are able to self-regulate and manage their emotions and control their behavior are better able to act in accordance with their values, manage stress, deal with conflict, persist in difficult times, see the good in others, and achieve their goals (Eisenberg, 2000; Boekaerts, 2011; Hofmann et al., 2014; Hampson et al., 2016). However, people with poor self-regulation skills may have problems with handling frustration and stress, and may lack self-esteem and self-confidence, which might result in anxiety and anger and, in the long term, in poor well-being, poor health and poor life conditions (Moffitt et al., 2011). Furthermore, poor self-regulation is predictive of antisocial behavior (Gardner et al., 2008) and externalizing problem behavior (Eisenberg, 2000; Oldehinkel et al., 2004).

Research has established that people differ in their capacity to regulate themselves and that the ability to self-regulate is an important factor in adolescents’ learning, cognitive and social functioning (Moffitt et al., 2011; Carlo et al., 2012; Hofmann et al., 2014; Dent and Koenka, 2016; Hampson et al., 2016; Panadero, 2017). It is not surprising that self-regulation (and its development) is important for adolescents’ functioning at school since during adolescence, academic learning becomes more difficult, and schooling becomes increasingly complex with multiple teachers, homework, and deadlines.

Several theories and models on self-regulation recognize the role of the context or the environment in the development of self-regulation (e.g., Pintrich, 2000; Järvelä and Hadwin, 2013; Zimmerman, 2013; Murray et al., 2015; de la Fuente-Arias, 2017; Panadero, 2017; and for an overview of theories, see Newman and Newman, 2020) and classroom studies suggest that characteristics of the social learning environment (including teachers’ behavior) have an impact on students’ self-regulation. However, most studies investigating these relations have a cross-sectional correlational design and do not relate to adolescents. As a result, there is little knowledge about relations and causal directions between context characteristics (e.g., referring to social learning environment, teacher behavior) and adolescents’ (development of) self-regulation. Since the ability to self-regulate is an important factor in adolescents’ learning, cognitive and social functioning, and also in their adult life, and neuroscience has demonstrated that during adolescence rapid changes in areas of the brain relevant for the ability to self-regulate are present (Blakemore and Choudhury, 2010; Luciana, 2010; Eldreth et al., 2013), which offers particular opportunities for interventions and indicates vulnerability and developmental plasticity for environmental influences, it is important to get a better understanding of which aspects of the learning environment that teachers help to create enhance and thwart adolescent students’ development of self-regulation. More in particular, longitudinal studies are needed that pay attention to characteristics of the learning environment and to teacher behavior in classes that is conducive and supportive to and not thwarting the development of adolescent students’ self-regulation.

## Theoretical background

### Self-regulation

In the literature on self-regulation, numerous, generally overlapping, conceptualizations can be found. For example, Gillebaart (2018) defines self-regulation, in line with Carver and Scheier (2012), as “the whole system of standards, thoughts, processes and actions that guide people’s behavior toward desired end states” (p. 3). These desired end states may be long-term goals, but can also be other standards or norms. It is closely related to the concept of self-control (Gillebaart, 2018), however, it involves more than controlling behavior since it provides “the entire scaffolding for successful goal pursuit” (Gillebaart, 2018, p. 3). According to Gillebaart (2018) self-regulation differs from self-control in that the ability to self-regulate “allows people to formulate goals, standards, and desired end-states, as well as to monitor any discrepancies between one’s current state and these desired end-states, whereas everything that one does to steer one’s behavior toward the desired end state constitutes self-control” (p. 3). Brown (1998, p. 62) defines self-regulation as people’s capacity to “plan, monitor and direct their behavior in changing situations” and stresses that people plan, monitor, assess and reflect on their own behavior on a regular basis and in periods of time. Together with Brown (1998) and de la Fuente-Arias (2017) considers self-regulation as the degree of a person’s positive proactivity … in his active and adequate management of the regulation of his conduct’ (p. 2). The process of self-regulation is influenced by many variables, pre-eminently control, self-efficacy, and motivation (Pintrich, 1999; Zimmerman, 2001, 2008; Pintrich and Zusho, 2002; Torrano and González-Torres, 2004; Baumeister and Vohs, 2007; Gardner et al., 2008; Bandura, 2012; Vancouver, 2018).

### Self-regulation related to learning: Self-regulated learning

In addition to self-regulation as a general construct, also constructs relating to particular domains can be found in the literature on self-regulation, for example, self-regulation constructs focusing on the regulation of emotions (Eisenberg and Spinrad, 2004; Boekaerts, 2011) or related to learning (Boekaerts, 1996, 2011; Pintrich, 2000; Winne and Hadwin, 2008; Efklides, 2011; Zimmerman, 2015; de la Fuente-Arias, 2017; Panadero, 2017; Schunk and Greene, 2018). In general, the term self-regulation, when applied to learning, refers to learners’ proactive process which consists of setting goals for their learning, actively monitoring their progress, and regulating their cognition, motivation, and behavior in order to achieve their learning goals (Pintrich, 2000). The term self-regulated learning is often used in this context. Although there are some variations in the definition of self-regulated learning in the literature, all definitions mention a direction towards goals and the use of self-regulation properties/strategies (de la Fuente-Arias, 2017). Furthermore, self-regulated learning is considered as a complex, dynamic, strategic, and cyclical process (Zimmerman, 2000, 2008; de la Fuente-Arias, 2017; Panadero, 2017) which consists of several phases (Pintrich, 2000; Zimmerman, 2000, 2008). As a construct, self-regulated learning is understood as a multidimensional construct referring to learners as active, goal-directed, strategic, and reflective individuals who plan, monitor, and regulate and reflect on their cognition, motivation, emotion/affect, and behavior to reach their desired goals (Pintrich, 2000; Panadero, 2017; Schunk and Greene, 2018). Numerous studies have established the importance of self-regulated learning to success in school and in further life (Zimmerman, 1990; Dent and Koenka, 2016; Artuch-Garde et al., 2017; Venitz and Perels, 2018; Jansen et al., 2019; Chu et al., 2020; Theobald, 2021).

A key component of self-regulated learning is the use of (self-regulated) learning strategies, and, in particular, the use of metacognitive strategies (Winne and Perry, 2000; Duckworth et al., 2011; Roelle et al., 2017). Learning strategies are self-initiated approaches to enhance learning (Zimmerman, 2015) and can refer to cognitive and metacognitive strategies. While cognitive strategies include students’ use of basic and complex strategies for the processing of information such as rehearsal, elaboration, and organization (Garcia and Pintrich, 1994, 1995), metacognitive strategies refer to strategies that learners can use to control and to regulate their own cognition and thinking processes. They include strategies such as planning, monitoring, and regulating learning (Garcia and Pintrich, 1994). It also includes reflecting on and evaluating the effectiveness of their learning approaches (Credé and Phillips, 2011). Research indicates a positive relationship between the use of self-regulated learning strategies and a variety of school outcomes including school performance (for an overview, see for example Tuero et al., 2022) and intervention studies proved that the use of these strategies is trainable (Núñez et al., 2021; see also meta-analyses of Dignath and Büttner, 2008; Dignath et al., 2008; Jansen et al., 2019; Theobald, 2021). Moreover, the use of metacognitive strategies seems to correlate, on average, stronger with school/academic performance than cognitive strategies do (Credé and Phillips, 2011; Dent and Koenka, 2016; Chow and Chapman, 2017) and intervention studies aiming at enhancing students’ self-regulated learning seem to be somewhat more effective in enhancing the use of metacognitive strategies than in enhancing the use of cognitive strategies (Theobald, 2021). Furthermore, there is evidence that older students (i.e., from secondary education on), benefit more from interventions including more metacognitive aspects (Dignath and Büttner, 2008; Panadero, 2017).

With regard to individual factors influencing students’ development of self-regulated learning and use of learning strategies, theory suggests and research has established that students’ emotions and beliefs about their own ability (self-efficacy, feelings of competence) play a key role (Pintrich, 1999; Pintrich and Zusho, 2002; Torrano and González-Torres, 2004; Pajares, 2008; Zimmerman and Cleary, 2009; Wigfield et al., 2011; Bandura, 2012) and that students with self-regulation learning skills are unlikely to use them proficiently if they have doubts about their learning capabilities (Duckworth et al., 2011).

### Development of self-regulated learning: The importance of learning context and teacher behavior

Self-regulated learning does not take place automatically (Winne, 2005) and there are some indications that students’ self-regulated learning often declines within the first year of secondary education (Van der Veen and Peetsma, 2009; Schuitema et al., 2012) and with increasing grade level (Vansteenkiste et al., 2009).

Winne (2005) stresses that students need support to become good self-regulated learners. Moreover, although self-regulated learning seems not easily be induced (Struyven et al., 2006) and it may take time to see the effectiveness of an intervention (Tuero et al., 2022; perhaps because it may take a while for students to adapt and alter their learning behavior patterns), there is clear evidence that students’ self-regulated learning is malleable and that, with adequate support and scaffolding, students can improve their self-regulated learning (Torrano and González-Torres, 2004; Dignath et al., 2008; Dignath and Büttner, 2008; Jansen et al., 2019; Theobald, 2021). This evidence suggests that students’ learning environments at school and in class matter. Attention for the importance of the (learning) context or environment, and, in particular, the teaching and the behavior of teachers in that context, is not new. For example, Zimmerman (1989) already mentioned ‘environment’ in his triadic model of self-regulated learning and also Pintrich (2000), Hadwin et al. (2011, 2018), and Järvelä and Hadwin (2013) acknowledge in their theoretical models that contextual features in the environment can guide and constrain students’ self-regulated learning. The importance of the learning environment and teachers’ teaching and behavior is also recognized in the recently formulated theory of de la Fuente-Arias (2017) on self- vs. externally regulated learning. In his theory, de la Fuente-Arias stresses “that self-regulated learning is dependent on external feedback, especially during situations of sustained effort and when goals must be maintained over time” (p. 3), and he acknowledges the importance of effective/regulatory teaching including, among others, “clearly defining tasks” (p. 5), “facilitating a context of personal involvement and persistence” (p.5), the promotion of self-control and self-observation (which includes the use of metacognitive strategies), and the promotion of self-reflection by means of adjusted feedback, dialog, and affective persuasion. Other researchers refer to optimal conditions for developing self-regulation and mention learning environments in which students get the opportunity to pursue goals that they themselves find meaningful and in which students are invited to develop their skills by selecting their own activities, by taking initiative, by engaging in challenging and collaborative learning experiences, and by making their own decisions (Fredricks et al., 2004; Boekaerts and Corno, 2005). Fredricks et al. (2004) mention in their review also the importance of a combination of academic and social support from the teacher and of offering structure (i.e., being clear about expectations), which is in line with findings from research studying the influence of caregivers (parents, teachers, mentors) on children’s ability to self-regulate. In this research, evidence is found for the importance of warm and responsive caregivers, the utilization of positive behavior management strategies, and the provision of a positive climate for growth and development in which caregivers provide support, coaching and modeling. Otherwise stated, these findings indicate the importance of caregivers’ co-regulation (Murray et al., 2015, 2016; Housman et al., 2018) and their creation of structured environments in which students/children have opportunities to practice with guidance (Murray et al., 2016). It is less clear, however, which characteristics of the learning environment (actively) constrain or undermine students’ development and engagement in self-regulated learning, and what explanatory mechanisms are involved. Knowledge of this may be important in explaining the often found decline in students’ self-regulation during secondary education. In addition, it is unclear how quickly this decline in self-regulation occurs after entering secondary education.

Furthermore, in addition to the relevance of “objective” characteristics of the learning environment, several theorists and researchers point to the importance of considering students’ perceptions of their learning environment and their teachers’ behavior (e.g., Reeve and Deci, 1996; Boekaerts and Niemivirta, 2000; Pintrich, 2000; Ryan and Patrick, 2001; Schuitema et al., 2012; Ryan and Deci, 2020). Reference is made, among others, to the perception of classroom norms (e.g., allowance of autonomy or control, autonomy support), perceived teacher support and structure, and classroom climate (including teacher warmth), and a plea is made for more research on how different features of the context can shape, facilitate, and constrain self-regulated learning.

### Self-determination theory and self-regulated learning

A theory that fits well with the concept of self-regulation and self-regulated learning as a form of optimal functioning and that addresses characteristics of the learning environment that can be useful pointers for discerning supportive vs. undermining/thwarting features of a learning environment in relation to students’ (development of) self-regulated learning, is the self-determination theory. In addition, this theory recognizes the importance of how students perceive their learning environment.

According to the self-determination theory (Ryan and Deci, 2000, 2020; Deci and Ryan, 2002), and in particular the sub-theory Basic Psychological Needs Theory—BPNT (Deci et al., 1996; Vansteenkiste et al., 2020; Opdenakker, 2021), students are more likely to engage in self-regulated learning if their learning environment satisfies their fundamental basic psychological needs, namely their need to feel autonomous, competent and related. When students feel autonomous, they act in congruence with their true selves. In addition, they express their genuine preferences in order to experience a general sense of choice, volition, willingness, and ownership. They experience a sense of integrity “as when their actions, thoughts, and feelings are self-endorsed and authentic” (Vansteenkiste et al., 2020, p. 3). Frustration of this need goes along with experiencing pressure, external control, conflict, or feeling pushed in a non-wanted direction (Ryan and Deci, 2020; Vansteenkiste et al., 2020). Feeling competent entails experiencing oneself as effective in interactions with the (social) environment, having opportunities to express and extend abilities, and feeling a sense of mastery (Deci and Ryan, 2002). When this need is frustrated, students feel personal ineffective and experience failure or helplessness (Vansteenkiste and Ryan, 2013; Vansteenkiste et al., 2020). Feeling related means feeling emotionally connected to others (Skinner and Pitzer, 2012), feeling loved and cared for, experiencing warmth, and feeling a sense of belonging (Baumeister and Leary, 1995). When this need is frustrated, students feel “a sense of social alienation, loneliness, and exclusion” (Vansteenkiste et al., 2020, p. 3). According to the self-determination theory, the social environment can support or thwart the mentioned needs leading to, respectively, growth, engagement, flourishing, and optimal functioning in case of supporting the needs (Deci and Ryan, 2002; Vansteenkiste et al., 2020) and malfunctioning when the mentioned needs are thwarted (Ryan and Deci, 2000).

Learning environments (and teachers) that are supportive to the three basic psychological needs are autonomy-supportive, deliver structure, and offer opportunities for feeling related and connected, for example, by means of an involved teacher (Opdenakker, 2021). Being an autonomy-supportive teacher entails that teachers take their students’ perspectives into account, acknowledge their feelings and perceptions, provide students with meaningful choices and allow them to make their own decisions about their learning (Deci et al., 1996; Williams and Deci, 1996). In addition, autonomy-supportive teachers help students to understand the relevance of learning tasks (Assor et al., 2002), give them explanatory rationales for engaging in requested endeavors, and allow them to act upon their personal values and interests in such a way that their learning is accompanied with a sense of volition and psychological freedom (Reeve, 2009; Opdenakker, 2021). This will stimulate students to engage in self-regulated learning (Deci and Ryan, 2000; Vansteenkiste et al., 2005). However, a learning environment in which high pressure and control is present and teachers make use of controlling language, students’ self-regulated learning will be thwarted (Deci and Ryan, 2000; Reeve, 2009).

Structure in the learning environment refers to offering informational and instructional support and supervision, guidance and help that meets students’ wishes and tries to overcome their problems. It further entails communication of clear expectations and presentation of clear goals, consistent guidelines, and rules so that students know what it takes to do well in class, and it also includes offering constructive feedback to students (Deci et al., 1996; Reeve, 2006; Vansteenkiste et al., 2012; Opdenakker, 2021). Structure primarily supports the need for competence and helps students to feel able to effectively deal with the learning task (Skinner and Belmont, 1993). A supportive, well-structured learning environment offers students optimal challenges and gives them opportunities for growth and for achieving success (Deci et al., 1996; Opdenakker, 2021). However, a learning environment characterized by confusion, vagueness and uncertainty, inconsistent teacher behavior, lack of help and competence-thwarting feedback, will thwart students’ self-regulated learning (Deci and Ryan, 2000).

Lastly, according to the self-determination theory, it is important that teachers create a caring, respectful, and supporting environment that meets students’ need for relatedness (Ryan and Deci, 2020; Opdenakker, 2021). The involvement of teachers and, in particular, their availability, genuine interest in their students, and their warm and caring presence is important in this respect (Deci et al., 1996; Deci and Ryan, 2000; Ryan and Deci, 2020; Opdenakker, 2021). In contrast, when teachers reject and neglect their students, their behavior is supposed to be a treat to the fulfillment of students’ basic psychological needs and can be seen as thwarting students’ basic psychological needs and, therefore, also their engagement in self-regulated learning (Deci and Ryan, 2000).

There is considerable evidence for the relevance and importance of the self-determination theory in education, linking effects of learning contexts (including teacher behavior) to students’ basic needs satisfaction and a variety of student/individual outcomes (for reviews, e.g., Ryan and Deci, 2000, 2020; Deci and Ryan, 2002; Vansteenkiste et al., 2020; Opdenakker, 2021; Conesa et al., 2022 ^1^). However, psychological need thwarting, that arises in response to perceiving that psychological needs are actively undermined, is understudied (Costa et al., 2015; Opdenakker, 2021). The few studies addressing this topic, found evidence for its relevance in relation to maladaptive functioning (see Bartholomew et al., 2018; Patall et al., 2018; Vandenkerckhove et al., 2019; Opdenakker, 2021; Conesa et al., 2022). Furthermore, there is some evidence that need-supportive teacher behavior is more important than need-thwarting teacher behavior for adaptive student behavior and optimal functioning (Skinner et al., 2008; Jang et al., 2016; Patall et al., 2018; Opdenakker, 2021) and that need-thwarting teacher behavior is more important for forms of maladaptive behavior and sub-optimal functioning (Jang et al., 2016; Patall et al., 2018; Opdenakker, 2021), although, depending on the student outcome, also unique and independent effects of both kinds of behaviors can be visible (Patall et al., 2018; Opdenakker, 2021).

### Links between supportive and thwarting teacher behavior and students’ self-regulated learning

Studies investigating effects of all mentioned supportive and thwarting teacher behavior dimensions on students’ (development of) self-regulated learning are scarce. Moreover, most of the studies exploring the link between teacher behavior and students’ self-regulated learning only address a selection of supportive teacher behavior, and studies exploring thwarting teacher behavior in combination with supportive teacher behavior are almost non-existent. A few exceptions are the study of Vansteenkiste et al. (2012), although this study strictly spoken rather focused on environments with high and low supportive teacher behavior, and the study of Opdenakker (2021). Vansteenkiste et al. (2012), explored effects of four perceived teaching configurations on self-regulated learning of secondary school students, namely configurations characterized by (1) (moderately high) autonomy support, (2) clear expectations (part of structure), (3) vague expectations and low autonomy support, and (4) high autonomy support and clear expectations. Their cross-sectional study revealed that the teacher configuration groups differed with regard to their students’ self-regulated learning: students in the teaching configuration characterized by (perceived) high autonomy support and clear expectations reported significant more self-regulated learning than students in the configuration with only autonomy support ore only clear expectations, and students in these groups had significant more self-regulated learning than students in het remaining group. Opdenakker (2021) investigated effects of perceived teachers supportive and thwarting behavior on secondary school students’ procrastination behavior (which is nowadays often considered as a self-regulation failure; see Steel, 2007). She found evidence for negative associations of the three mentioned teacher behavior support dimensions (autonomy support, structure, involvement) and evidence for positive associations of the mentioned teacher behavior thwart dimensions, indicating that teachers’ need-supportive behavior is associated with low procrastination behavior, while teachers’ need-thwarting behavior is associated with higher levels of procrastination behavior.

Furthermore, studies focusing on the association between dimensions of supportive teacher behavior and students’ (development of) self-regulated learning have demonstrated a positive relation between these dimensions and students’ self-regulated behavior. For example, Sierens et al. (2009) studied the relation between perceived teachers’ autonomy support, structure and self-regulated learning of secondary education students and found that structure was associated with more self-regulated learning under conditions of moderate and high autonomy support only. Also, Mouratidis et al. (2013) found evidence for the importance of structure in relation to self-regulated learning and their study revealed that this effect was partially mediated by competence need satisfaction. Schuitema et al. (2012) addressed the relationship between autonomy support, relevance (an aspect of autonomy support) and grade-7 students’ development of self-regulated learning and found positive effects of autonomy support (relevance) on aspects of self-regulated learning (metacognitive strategy use, delay of gratification). Schuitema et al. (2016) investigated in their longitudinal study the direction of the effects between students’ perceptions of teachers’ autonomy support and involvement on students’ self-regulated learning (metacognitive strategy use, delay of gratification) during their first 2 years in secondary education. They found that (only) perceived teachers’ involvement predicted (both aspects of) self-regulated learning. In addition, their study revealed small reciprocal effects in both directions between delay of gratification and perceived autonomy support and they found that metacognitive strategy use predicted perceived autonomy support. Yin et al. (2009) explored the association between teacher support (including aspects of teacher involvement) and aspects of students’ self-regulated learning. Their study also revealed links between teacher support and students’ self-regulated learning.

## Aim of the present study

In sum, it can be concluded that that teachers’ supportive behavior, as defined by self-determination theory, is positively related to students’ self-regulation related to learning (self-regulated learning). Furthermore, there is some indication that teacher behavior that is thwarting students’ basic psychological needs is harmful for self-regulation (self-regulated learning). However, as a result of the largely lack of studies that consider both supportive and undermining teacher behaviors in relation to students’ self-regulated learning, it is unclear how effects of supportive vs. undermining teacher behaviors relate to each other. Therefore, it is still unclear on which teacher behaviors interventions should focus (stimulating supportive behavior only and/or focusing on diminishing undermining behavior) to foster students’ development and engagement in self-regulated learning and to avoid a decline in students’ development and engagement in self-regulated learning. In addition, since most previous studies are cross-sectional, it is difficult to build knowledge on the causal directions of the relations between perceived teacher support, thwart and self-regulated learning. Furthermore, it is unclear how quickly the decline in self-regulation related to learning occurs in the first year of secondary education and if that decline is associated with students’ experiences and perceptions of their teachers’ supportive or thwarting behavior. The present study aims to contribute to reducing this knowledge gap and extends existing research by examining and comparing effects of a selection of perceived teacher supportive and undermining behavior (based on self-determination theory) on early adolescents’ self-regulation (self-regulated learning) within a longitudinal design in which students developments are followed from start of secondary education during their first months in their first year of secondary education.

## Materials and methods

### Participants

In the study, which is part of a larger research project on students’ motivational and self-regulated development during the first year of secondary education,^2^ 566 grade-7 students (55% boys, 45% girls) participated. They belonged to 20 mathematics/English secondary education classes of three public schools in the Netherlands which were located in a provincial city area in the northern part of the country. The schools were representative of typical public schools for middle socioeconomic status and voluntary participated in the research. Class sizes ranged from 21 to 31 students (*M* = 28, *Mdn* = 29, *SD* = 2.9), and half of the classes were English classes. The choice for Math and English classes is based on the importance and diversity of these subjects in grade 7 and because it was expected that choosing for these classes would result in heterogeneous teacher behavior. Classes of all school tracks of the regular Dutch education system were represented for both subjects: so-called transition classes (that combined several track levels in one class, 40%) were included as well as single-track classes (prevocational, general, and pre-university). Almost all students were native Duch (<1% was nonnative Dutch). The students’ mean age was 12.19 years (SD = 0.55) at the start of the school year.

### Procedure

Paper-and-pencil questionnaires were used to tap students’ self-regulation related to learning (self-regulated learning) at the start of the school year and after about 2 months. Students’ perceptions of their teachers’ need-supportive and need-thwarting behavior during the first months of the school year were collected with an additional paper-and-pencil questionnaire. The questionnaires were distributed during class time and permission to distribute them was received from the school authority as well as by means of written informed consent from the students’ math/English teachers and their parents/representatives. Students received an explanation of the purpose of the research before completing the questionnaires. They were assured of their confidentiality and anonymity, and in order to assure this, the administration of the questionnaires at the different time points was carried out by research assistants.

### Measures

#### Self-regulated learning

Self-regulated learning was assessed by means of an important aspect of self-regulated learning, namely the use of metacognitive strategies. A shorted Dutch validated version (with 6 items) of the use of metacognitive strategies scale of the MSLQ of Pintrich and De Groot (1990), the most used established instrument to measure self-regulated learning (Roth et al., 2016), was used. The scale measures the use of activities such as planning and comprehension monitoring. An example of an items is: “When I’m reading, I stop once in a while and go over what I have read.” Previous research has confirmed the reliability and validity of this instrument and indicates that it measures the same construct over time (Van der Veen and Peetsma, 2009; Schuitema et al., 2016). Cronbach’s α values are 0.63^3^ (start math/English) and 0.75/0.76 (second measurement, respectively math and English).

#### Need-supportive and need-thwarting teacher behavior

Students’ perceptions of their teachers’ need-supportive and need-thwarting behavior are assessed with The Questionnaire-on-Teacher-Support-and-Thwart (Opdenakker, 2021). The scales are based on ideas of the self-determination theory in which a distinction is made between dimensions that support vs. thwart three basic psychological needs. Supporting autonomy, delivering structure, and being involved with the students as a teacher are assumed to be important for the fulfillment of students’ basic psychological needs and are measured as individual scales. Exhibiting controlling (instructional) behavior, having chaos, uncertainty, and inconsistency in the classroom, and rejecting and neglecting students, are supposed to be a treat to the fulfillment of students’ basic psychological needs and are measured as individual scales as well. The questionnaire is based on the ‘Teacher as a Social Context’ (TASC; Belmont et al., 1992) and comprises 51 items referring support (autonomy support, structure, and teacher involvement), omission of support, and supposed opposites like controlling instructional behavior, chaos/uncertainty/inconsistency in the classroom, and teacher neglect/rejection. The items are clustered into six scales: three supporting and three thwarting scales. For convenience, we will refer to the dimensions/scales as autonomy support vs. teacher thwart—control, structure vs. teacher thwart—chaos/inconsistency, and teacher involvement vs. teacher thwart—neglect/rejection. The number of items of the six individual teacher behavior scales ranges from 5 to 12. Items were presented on a five-point Likert scale ranging from 1 = “strongly disagree” to 5 = “strongly agree.” Examples of the items are: “My teacher gives me a lot of choices about how I do my schoolwork” (autonomy support), “This teacher tries to control everything I do” (teacher thwart—control), “This teacher shows how to solve problems for myself” (structure), “My teacher keeps changing how he/she acts toward me” (teacher thwart—chaos/inconsistency), “This teacher really cares about me” (teacher involvement), and “My teacher does not seem to enjoy having me in his/her class” (teacher thwart—neglect/rejection). The psychometric properties of the individual scales are sufficient to good (Cronbach’s α values vary between 0.61 and 0.82).

### Method of analysis

In addition to descriptive statistics related to all variables and a correlation analysis related to corresponding teacher need-supportive and need-thwarting behavior, multilevel analyses (MLwiN; Rasbash et al., 2012) were performed to study the effects of the need-supportive and need-thwarting dimensions of teacher behavior on students’ self-regulated learning. Two levels were distinguished in the multilevel models, namely the class level (classes) and the student level (students within classes). In addition, self-regulated learning measured at the start of the school year was controlled for. A series of hierarchical models with and without a combination of (corresponding) need-supportive and need-thwarting teacher behaviors were inspected in order to explore evidence for unique and joint effects of these teacher behavior dimensions, or otherwise stated, to explore total effects and evidence for additional effects of teacher behavior dimensions. A selection of these models, of which the results provide a comprehensive overview of the findings, will be presented in a table. In accordance with usual practice, results in the tables are presented with significance levels referring to two-sided testing. However, based on the literature/theoretical framework (and expectations derived from it), one-sided testing is allowed with regard to the effects of teaching behavior.

## Results

### Descriptive statistics

In Table 1, the descriptive statistics of all variables (means and standard deviations) are provided. The comparison of students’ self-regulated learning (use of metacognitive strategies) at start of the school with their self-regulated learning after about 2 months indicates that students’ self-regulated learning seems, in general, to decrease a little during that period (if this decreasing trend continues in a linear manner during the school year, then the decrease of self-regulated learning from start to the end of the school year is comparable to a small-to-medium effect size; Cohen, 1988; Lakens, 2013). In addition, inspection of the standard deviations of the indicator of self-regulated learning on the two occasions reveals there are clear indications of differences between students with regard to the degree they learn self-regulated, and in particular, the degree they make use of metacognitive strategies on the two measured occasions. In addition, these differences between students seem to increase during the school year.

**Table 1:** Means (M) and standard deviations (SD) for students’ self-regulated learning and teacher support and teacher thwart dimensions.

	*M*	*SD*
**Student self-regulation**		
Self-Regulation (start)	3.23	0.59
Self-Regulation (end)	3.19	0.70
**Support and thwart dimensions**		
Autonomy support	3.20	0.70
Teacher thwart—Control	2.04	0.62
Structure	3.48	0.67
Teacher thwart—Chaos/inconsistency	1.95	0.58
Teacher involvement	3.02	0.64
Teacher thwart—Neglect/rejection	1.70	0.66

With regard to students’ perception of their teachers’ need-supportive and need-thwarting behavior, grade-7 students score their teachers’ behavior, on average, as more need-supportive than need-thwarting. Furthermore, they score “giving structure,” on average, as highest of their teachers’ supportive behaviors and score the supportive teacher behavior “being involved as a teacher” as lowest, although the score on the last-mentioned dimension is still at the middle of the rating scale. Of the need-thwarting behaviors, students score their teachers highest on “exhibiting controlling behavior” and on “having chaos, uncertainty and behaving inconsistent toward them.” However, these scores are, on average, one point lower than the middle of the rating scale. In addition, the table reveals that students perceive differences in their classes with regard to their teachers’ behaviors.

As mentioned, also a correlational analysis related to corresponding need-supportive and need-thwarting teacher behavior dimensions was conducted. This analysis revealed that the correlations between the support and thwart dimensions ranged from −0.39 (teacher involvement and teacher thwart—neglect/rejection) to −0.55 (structure and teacher thwart—chaos/inconsistency), with the correlation between autonomy support and teacher thwart—controlling behavior being −0.51. These correlations between corresponding dimensions indicate a rather modest covariance, and implicate that, although there is ground for common variance, there are also clear indications that these dimensions measure unique parts of teacher behavior.

### Main analysis

Multilevel analyses with the teacher dimensions separately included in the multilevel model revealed that both (perceived) need-supportive and need-thwarting teacher behaviors could explain differences (and changes) in students’ self-regulation. The results indicated that the development of students’ self-regulation was positively related to autonomy support, delivering structure, and having a teacher who is involved with students. In addition, when teachers thwarted their students’ basic psychological needs, this negatively affected early adolescents’ self-regulation. Furthermore, the degree to which the teacher delivered structure seemed to be the most important supportive dimension, followed by the degree of the involvement of the teacher toward their students. The degree of autonomy support was important as well, but to a lesser extent. With regard to the need-thwarting dimensions, controlling teacher behavior as well as the creation of a chaotic learning environment with uncertainty and inconsistent teacher behavior seemed to be the most harmful teacher behaviors, with, to a clear lesser extent, also teacher behavior characterized by neglecting and rejecting students. With regard to the total effects of the teacher behavior dimensions on students’ self-regulated learning, need-supportive teacher behavior dimensions explained between 9% and 15% of the variance in students’ self-regulation and need-thwarting teacher dimensions between 3% and 7%.

A further inspection of the results comparing effects of supportive vs. thwarting teacher behavior revealed that teachers’ supportive behaviors was stronger related to students’ self-regulated learning development compared to teachers’ thwarting behavior. Moreover, additional analyses in which corresponding supportive and thwarting teacher behavior dimensions are included together in the same multilevel model (see Table 2), revealed that all teacher supportive dimensions remained to have significant positive effects on students’ self-regulated learning, but that of the thwarting dimensions only the effect of controlling (instructional) teacher behavior remained significant in addition to the corresponding teacher supportive dimension of autonomy support. These results indicate the supremacy of all supportive teacher behavior dimensions in relation to students’ (development) of self-regulated learning compared to thwarting teacher dimensions and, in addition, deliver evidence for the harmful effects of controlling instructional teacher behavior on the development of early adolescent students’ self-regulation related to learning after students’ transition from primary to secondary education and during the first months of their first year in secondary education.

**Table 2:** Results of multilevel models explaining students’ self-regulated learning by teacher support and thwart dimensions and self-regulated learning at the start of the school year.

	Involvement—Neglect/rejection (*N* = 539)		Structure—Chaos/inconsistency (*N* = 541)		Autonomy—Control (*N* = 530)	
	Coefficient	SE	Coefficient	SE	Coefficient	SE
**Fixed effect**						
Intercept	3.209^**^	0.041	3.208^**^	0.042	3.199^**^	0.039
Teacher support	0.284^**^	0.048	0.341^**^	0.048	0.150^**^	0.046
Teacher thwart	0.023	0.046	0.036	0.055	−0.096^°^	0.052
Self-regulated learning (start)	0.357^**^	0.046	0.336^**^	0.046	0.373^**^	0.048
**Random effect**						
Level 2 variance	0.020	0.011	0.022	0.011	0.016	0.010
Level 1 variance	0.378	0.023	0.360	0.022	0.387	0.024
Deviance	1022.535		1002.476		1019.672	

°*p* < 0.10,

* *p* < 0.05,

** *p* < 0.01 (two-sided testing).

## Conclusions and discussion

The present study aimed to contribute to the knowledge base on the effects of (perceived) need-supportive and need-thwarting teacher behavior on early adolescents’ self-regulation, and in particular, on students’ self-regulated learning during their first months in secondary education By addressing need-supportive teacher behavior as well as need-thwarting teacher behavior within the same study, the study was quite unique, since almost no previous study addressed all supportive and thwarting teacher behaviors based on self-determination theory within the same study (in relation to the self-regulation of students). Also, the application of a longitudinal design while investigating effects of teacher behavior in accordance with the self-determination theory is rather scarce in ecological valid environments, and, as such, the present study extends existing research as well.

### Main conclusions

In this study, evidence was found for positive effects of need-supportive teacher behavior and for negative effects of need-thwarting teacher behavior on early adolescents’ development in self-regulated learning during their first months in secondary education. However, the effects of need-supportive teacher behavior were stronger than the effects of need-thwarting behavior. These findings are in line with the self-determination theory, and in particular the sub-theory basic psychological needs theory (Vansteenkiste and Ryan, 2013), and with the (scarce) research literature on effects of need-supportive and need-thwarting teacher behavior on optimal functioning and adaptive student behavior (Skinner et al., 2008; Jang et al., 2016; Patall et al., 2018; Ryan and Deci, 2020; Vansteenkiste et al., 2020; Opdenakker, 2021). In addition, the findings regarding the importance of supportive teacher behavior on students’ self-regulated learning are in agreement with findings regarding the influence of social environments and caregivers (e.g., parents, teachers) on children’s (development of) self-regulation (Murray et al., 2015).

Furthermore, the results showed that need-thwarting teacher behavior can have unique and independent effects (in addition to joint effects with need-supporting behavior) on the development of students’ self-regulated learning (which can be considered as a form of optimal functioning). More in particular, it was found that controlling teacher behavior had a unique (negative) effect on students’ (development of) self-regulated learning in addition to the (positive) effect of teachers’ autonomy-supportive behavior. Finding evidence for unique effects of need-thwarting teacher behavior on optimal functioning and adaptive student behavior in addition to clear effects of need-supportive teacher behavior, is in agreement with the scare literature on this topic (Patall et al., 2018; Opdenakker, 2021) and self-determination theory (Vansteenkiste and Ryan, 2013). In addition, it provides additional evidence for the importance of paying attention not only to supportive teacher behavior, but also to undermining or thwarting teacher behavior, which is also advocated by Costa et al. (2015) and Vansteenkiste et al. (2020), since it does yield, in some cases, additional functional costs (Vansteenkiste et al., 2020).

With regard to effects of supportive teacher behavior dimensions on students’ (development of) self-regulated learning, all dimensions (structure, autonomy support, teacher involvement) had clear significant positive effects. However, there were also differences in the size of the effects, with structure having the largest effect, followed by teacher involvement, and autonomy support having the least strong effect. In the literature on effects of supportive teacher behavior (defined in line with the self-determination theory) on self-regulated learning, also positive effects are found for structure (Sierens et al., 2009; Vansteenkiste et al., 2012; Mouratidis et al., 2013), teacher involvement (Schuitema et al., 2016), autonomy support (Schuitema et al., 2012; Vansteenkiste et al., 2012), and teacher support (Yin et al., 2009). However, not many studies addressed effects of all three mentioned need-supportive teacher behavior dimensions together in the same research on students’ self-regulated learning, which makes it difficult to compare the results of the present study regarding the importance level of the dimensions with findings in the literature. A few studies addressed autonomy support as well as structure in relation to self-regulated learning and found evidence for the importance to combine structure with autonomy support (Sierens et al., 2009; Vansteenkiste et al., 2012). In fact, Sierens et al. (2009) found only a significant (unique) effect of structure and not of autonomy support, which seems to be in line with the finding in the present study that the effect of structure is larger than that of autonomy support. Also, Schuitema et al. (2016), who also investigated effects of both dimensions but included only one teacher behavior dimension in their cross-lagged models at the same time, found no significant effect of autonomy support on self-regulated learning (use of metacognitive strategies) when the results of their final cross-lagged model are considered. Interesting was that Sierens et al. (2009) also modeled an interaction between structure and autonomy support and discovered that structure was associated with more self-regulated learning (only) when it was accompanied with moderate and high autonomy support. The importance of structure in developing students’ self-regulated learning is also in line with intervention research aimed at fostering self-regulated learning of students. In this research, structured environments in which students receive strategy instruction, support, and opportunities to practice with the use of strategies seem to be fruitful and are highly advocated (Torrano and González-Torres, 2004; Dignath et al., 2008; Dignath and Büttner, 2008; Jansen et al., 2019; Theobald, 2021). In addition, the idea of scaffolding, which is one of the most utilized instructional strategies in these interventions (Torrano and González-Torres, 2004) and entails delivering support to students while they are learning (self-regulation strategies) and eliminating this support step-by-step over time as students become more competent, is quite in congruence with the findings of the present study and previous studies (investigating effects of environment dimensions in line with self-determination theory). It also underscores importance of delivering structure while helping students to become autonomous learners, and, thus, support students’ autonomy. Furthermore, the finding that structure and autonomy support are both important and, that for an optimal learning environment, structure should be accompanied with medium to high levels of autonomy support (Sierens et al., 2009) could deliver an explanation (in addition to individual developmental characteristics of students) for the often-found decrease (or at most stability) in students’ self-regulated learning during secondary education. In the present study, students perceived their teachers’ behavior with regard to delivering structure and offering autonomy support as being at most as medium, indicating that, on average, they did not really experience very optimal teacher behaviors and a learning environment optimal for the development of (and engagement in) self-regulated learning. Furthermore, the idea of the importance of both structure and autonomy support is also in line with the recently formulated theory of de la Fuente-Arias (2017) of self- vs. externally-regulated learning™ and is congruent with recent empirical and theoretical work on motivating and demotivating teaching styles using a circumplex approach (Aelterman et al., 2019; Vermote et al., 2020; Moè et al., 2022).

A remarkable finding was the importance of teacher involvement in the present study. Teacher involvement seemed to be the second most important teacher behavior in relation to students’ development of self-regulated learning. This means that students must feel cared for by their teachers, must feel that their teachers are interested in them, must experience sincere concern and responsiveness from their teacher to them in order to develop and engage in self-regulated learning. Also, Schuitema et al. (2016) found evidence for the importance of teacher involvement on students’ self-regulated learning (use of metacognitive strategies, delay of gratification) in their longitudinal study and this finding is in line with research studying the influence of caregivers (parents, teachers, mentors) on children’s ability to self-regulate and to engage in academic learning pointing to the importance of warm and responsive caregivers (Stroet et al., 2013; Murray et al., 2015, 2016; Housman et al., 2018; Opdenakker, 2020, 2021). According to the self-determination theory, teachers’ involvement with their students plays an important role in students’ internalization process by which they ‘attempt to transform socially sanctioned mores or requests into personally endorsed values and self-regulations’ (Deci and Ryan, 2000, p. 235–236). Involved teachers are supportive for students’ need to feel related and competent (and autonomous) and thereby facilitate the internalization of values and regulations (Deci and Ryan, 2000). By satisfying students’ need for belongingness or relatedness, involved teachers nurture the motivational basis for internalization, ensuring a more effective transmission of values and regulations and a more cohesive social (class) organization (Deci and Ryan, 2000). In addition to the self-determination theory, also theories (e.g., attachment theory, teaching through interactions framework, model of interpersonal teacher behavior) and classroom studies addressing the influence of social (learning) environments on student learning and student (motivational) outcomes stress and, in case of research studies, have repeatedly demonstrated the importance of having warm teacher-students relationships (Opdenakker et al., 2012; Stroet et al., 2013, 2015; Sparks et al., 2016) and creating a warm and safe climate in classes for students’ learning (for a discussion of theories and research, see Opdenakker, 2020, 2022; Opdenakker and Van Damme, 2006a,b, 2007). Teachers who are involved with their students are able to create such environments (Opdenakker and Van Damme, 2006b; Fraser, 2012; Opdenakker, 2020, 2022).

In sum, the present study was among the first to investigate the effects of three dimensions of need-supportive teacher behavior and three dimensions of need-thwarting teacher behavior in line with SDT-BPNT within the same study and to explore the effects of these teacher behavior dimensions while adopting a longitudinal design. The findings revealed that this approach is a fruitful way to gain insights into students’ development of and engagement in self-regulated learning and highlighted the importance of delivering structure, being involved as a teacher and providing autonomy support to students to stimulate their engagement in and development of self-regulated learning. However, the findings also demonstrated harmful effects of teachers’ controlling behavior in addition to the positive effects of teachers’ autonomy supportive behavior (even within the first months of students’ first year in secondary education).

### Limitations and suggestions for further directions

Although the present study expanded existing research and revealed important results of teacher behavior effects on early adolescents’ self-regulated learning in line with self-determination theory, the study has also a number of limitations.

The first limitation is that students’ self-regulation was solely studied in relation to the domain of learning and, within that domain, limited to the use of meta-cognitive strategies. It is possible that when other aspects of self-regulated learning or self-regulation are studied (such as delay of gratification, emotion regulation or effort regulation), different findings with regard to the relative importance of the explored need-supportive and need-thwarting teacher behaviors are found. However, based on the study of Schuitema et al. (2016), in which two aspects of self-regulated learning (use of metacognitive strategies and delay of gratification) are addressed, the differences in results seem to be rather minor. It is possible, however, that when self-regulation in different domains are compared, the differences in findings are larger. Research of Murray et al. (2022) is interesting in this respect. The findings of their meta-analysis suggest not only that emotion regulation may be a critically important self-regulation mechanism during early adolescence, but also that intervention approaches focusing predominantly on emotion regulation seem to significantly improve behavioral as well as emotional outcomes. More research is needed to explore whether the hierarchy of effects found in the present study are also valid when emotion regulation is the subject of research. Moreover, it will be interesting to pay in particular attention to aspects of co-regulation between teachers and students, which is rather understudied in relation to students’ emotion regulation (Murray et al., 2016).

Secondly, the reliance on student perceptions of teacher behavior and on self-reports of self-regulated learning could be seen as another limitation. Although student perceptions of their teachers’ behavior are seen as very valuable and convenient in learning environment research (Opdenakker, 2021, 2022) and within the perspective of the self-determination theory (Ryan and Deci, 2020), and have high validity (Kulik, 2001), using student ratings for teacher behavior as well as for students’ self-regulated learning might have inflated the observed associations between them because of shared method variance. In addition, it might be that students’ ability to regulate their own learning at the start of the school year has an effect on the way they perceive their teachers’ behavior during the school year. The addition of observational data and interviews with students about how they perceive their teachers’ behavior could be of added value to gain more complementary and deeper insights in future research.

A third limitation of the present study is that, although a longitudinal approach was used, it was not possible to study reciprocal effects between teacher behavior and students’ self-regulated learning because students’ self-regulated learning was measured only twice and teacher behavior only once. Since there are some indications in the literature that students’ behavior (and the way in which their behavioral is regulated, i.e., autonomous or controlled motivated) can influence (need-supportive) teacher behavior (and vice versa; see studies of Skinner and Belmont, 1993, Jang et al., 2016, Schuitema et al., 2016, Matos et al., 2018, and Garn et al., 2019), and more in particular, that aspects of students’ self-regulated learning such as delay of gratification predicts perceived autonomy support (and vice versa; Schuitema et al., 2016), it is of importance to pay attention to reciprocal effects of teacher behavior and students’ self-regulated learning in future longitudinal studies. This will imply that longitudinal studies should be designed with enough/more measurement points and appropriate time intervals between them. Based on their study, Schuitema et al. (2016) suggest that these intervals should be smaller than half a school year to get more insight into how dynamic variables such as teacher behavior and students’ self-regulated learning influence each other over time, which is in agreement with the recommendation of Collins and Graham (2002) to use shorter time intervals in longitudinal studies investigating influences between dynamic variables. In addition, it is relevant to focus not only on teachers’ need-supportive behavior in these studies, but also to need-thwarting teacher behaviors, since there are indications in recent research that student behavior can not only effect teachers’ need-supportive behavior, but also their undermining behavior (see Jang et al., 2016).

Despite the mentioned limitations, the findings of the present study contribute to our knowledge and growing understanding of the influences of learning environments and, more in particular, of facilitating and undermining factors in these environments (teacher behavior) in relation to (adolescent) students’ development and engagement in self-regulated learning. The findings contribute to highlighting the importance of both teachers’ need-supportive and need-thwarting behaviors in daily classrooms and indicate that, in particular, need-supportive teacher behavior (structure, teacher involvement, and autonomy support) should be fostered and controlling teacher behavior should be avoided when the development of students’ self-regulated learning and students’ engagement in this kind of behavior is focused on. Furthermore, the results point to the relevance of paying attention to the delivery of structure when adolescent students’ development and engagement in self-regulated learning is considered. This seems, at first side, somewhat counterintuitive since students’ self-regulation and autonomy is highly important in self-regulated learning. However, it is important to consider that the study was conducted with students in their first year of secondary education and started when these students had made the transition from primary school to secondary school. In addition, the findings are in line with instructional approaches such as scaffolding, that has proven to be effective in supporting students to become self-regulated learners, and are also in agreement with ideas of co-regulation and findings from research based on self-determination theory that emphasize the relevance of combining autonomy-support and delivering structure to students. The study also shows the relevance of being involved with students as a teacher. For students’ development and optimal functioning, but also for the internalization of values and regulations, it is known from theory, research, and practice that it is important that students feel cared for by their teachers, that they feel that their teachers are interested in them, have sincere concern and are responsive to them. Although further research is necessary, since this teacher need-supportive dimension is rather understudied in comparison to the other need-supportive dimensions within research based on self-determination theory, and also in relation to self-regulated learning, the present study indicates that the quality of the relation teachers have with their students matters not only for students’ social and emotional development and well-being (as is clearly demonstrated in much research from a developmental psychology perspective), but matters also for their learning behavior, which is important for their performance and success at school and for their success in later life.

## Data availability statement

The raw data supporting the conclusions of this article will be made available by the authors, without undue reservation.

## Ethics statement

Ethical review and approval was not required for the study on human participants in accordance with the local legislation and institutional requirements. Written informed consent to participate in this study was provided by the participants’ legal guardian/next of kin.

## Author contributions

M-CO designed the study, was in charge of the data collection procedure, analyzed the data, and wrote the manuscript.

## Conflict of interest

The author declares that the research was conducted in the absence of any commercial or financial relationships that could be construed as a potential conflict of interest.

## Publisher’s note

All claims expressed in this article are solely those of the authors and do not necessarily represent those of their affiliated organizations, or those of the publisher, the editors and the reviewers. Any product that may be evaluated in this article, or claim that may be made by its manufacturer, is not guaranteed or endorsed by the publisher.

## Footnotes

^1^The review of Conesa et al. (2022), however, revealed that evidence of its influence (in terms of evidence for the importance of basic psychological need satisfaction) in primary education classrooms is still limited due to the lack of studies (that contain rigorous methodology). It must be said, however, that Conesa et al. (2022) excluded studies conducted in a physical education context from their review.

^2^The study of Opdenakker (2021) is also part of this research project.

^3^According to Sijtsma (2009), Cronbach’s alpha should be considered as one of the smallest lower bound estimates of reliability. In addition, its values are quite sensitive to the number of items in the scale (Nunnally, 1978). With short scales (e.g., scales with fewer than 10 items), it is common to find quite low Cronbach values (e.g., 0.5; Pallant, 2011). In this case, Briggs and Cheek (1986) suggest reporting also the mean inter-item correlation for the items and recommend an optimal range for the mean inter-item correlation between 0.2 and 0.4. In the case of the self-regulated learning measures at the start of secondary education, the mean inter-item correlation was 0.22, which is within the mentioned optimal range.

## References

[ref1] Aelterman N. Vansteenkiste M. Haerens L. Soenens B. Fontaine J. R. Reeve J. (2019). Toward an integrative and fine-grained insight in motivating and demotivating teaching styles: the merits of a circumplex approach. J. Educ. Psychol. 111, 497–521. doi: 10.1037/edu0000293

[ref2] Artuch-Garde R. González-Torres M. C. de la Fuente J. Vera M. M. Fernández-Cabezas M. López-García M. (2017). Relationship between resilience and self-regulation: a study of Spanish youth at risk of social exclusion. Front. Psychol. 8:612. doi: 10.3389/fpsyg.2017.00612, PMID: 28473792 PMC5397523

[ref3] Assor A. Kaplan H. Roth G. (2002). Choice is good, but relevance is excellent: autonomy enhancing and suppressing teacher behaviours predicting students’ engagement in schoolwork. Br. J. Educ. Psychol. 72, 261–278. doi: 10.1348/000709902158883, PMID: 12028612

[ref4] Bandura A. (2012). On the functional properties of perceived self-efficacy revisited. J. Manag. 38, 9–44. doi: 10.1177/0149206311410606

[ref5] Bartholomew K. J. Ntoumanis N. Mouratidis A. Katartzi E. Thøgersen-Ntoumani C. Vlachopoulos S. (2018). Beware of your teaching style: a school-year long investigation of controlling teaching and student motivational experiences. Learn. Instruct. 53, 50–63. doi: 10.1016/j.learninstruc.2017.07.006

[ref6] Baumeister R. F. Leary M. R. (1995). The need to belong: desire for interpersonal attachments as a fundamental human motivation. Psychol. Bull. 117, 497–529. doi: 10.1037/0033-2909.117.3.497, PMID: 7777651

[ref7] Baumeister R. F. Vohs K. D. (2007). Self-regulation, ego depletion, and motivation. Soc. Pers. Psychol. Compass 1, 115–128. doi: 10.1111/j.1751-9004.2007.00001.x

[ref8] Belmont M. Skinner E. Wellborn J. Connell J. (1992). Teacher as social context (TASC). Two measures of teacher provision of involvement, structure and autonomy support. Technical Report. New York, NY: University of Rochester.

[ref9] Blakemore S.-J. Choudhury S. (2010). Development of the adolescent brain: implications for executive function and social cognition. J. Child Psychol. Psychiatry 47, 296–312. doi: 10.1111/j.1469-7610.2006.01611 16492261

[ref10] Boekaerts M. (1996). Self-regulated learning at the junction of cognition and motivation. Eur. Psychol. 1, 100–112. doi: 10.1027/1016-9040.1.2.100

[ref11] Boekaerts M. (2011). “Emotions, emotion regulation, and self-regulation of learning,” in Handbook of self-regulation of learning and performance. eds. Zimmerman B. J. Schunk D. H. (New York/London: Routledge/Taylor & Francis Group), 408–425.

[ref12] Boekaerts M. Corno L. (2005). Self-regulation in the classroom: a perspective on assessment and intervention. Appl. Psychol. 54, 199–231. doi: 10.1111/j.1464-0597.2005.00205.x

[ref13] Boekaerts M. Niemivirta M. (2000). “Self-regulated learning: finding a balance between learning goals and ego-protective goals,” in Handbook of self-regulation. eds. Boekaerts M. Pintrich P. R. Zeidner M. (San Diego, CA: Academic Press), 417–451.

[ref14] Briggs S. R. Cheek J. M. (1986). The role of factor analysis in the development and evaluation of personality scales. J. Pers. 54, 106–148. doi: 10.1111/j.1467-6494.1986.tb00391.x

[ref15] Brown J. M. (1998). “Self-regulation and the addictive behaviors,” in Treating addictive behaviors. 2nd Edn. eds. Miller W. R. Heather N. (New York, NY: Plenum Press), 61–73.

[ref16] Carlo G. Crockett L. J. Wolff J. M. Beal S. J. (2012). The role of emotional reactivity, self-regulation, and puberty in adolescents’ prosocial behaviors. Soc. Dev. 21, 667–685. doi: 10.1111/j.1467-9507.2012.00660.x, PMID: 28316370 PMC5356224

[ref17] Carver C. S. Scheier M. F. (2012). Attention and self-regulation: A control-theory approach to human behavior. Berlin: Springer.

[ref18] Chow C. W. Chapman E. (2017). Construct validation of the motivated strategies for learning questionnaire in a Singapore high school sample. J. Educ. Dev. Psychol. 7, 107–123. doi: 10.5539/jedp.v7n2p107

[ref19] Chu L. Li P.-H. Yu M.-N. (2020). The longitudinal effect of children’s self-regulated learning on reading habits and well-being. Int. J. Educ. Res. 104:101673. doi: 10.1016/j.ijer.2020.101673

[ref20] Cohen J. (1988). Statistical power analysis for the behavioral sciences (2nd ed.). New York, NY: Routledge Academic.

[ref21] Collins L. M. Graham J. W. (2002). The effect of the timing and spacing of observations in longitudinal studies of tobacco and other drug use: temporal design considerations. Drug Alcohol Depend. 68, 85–96. doi: 10.1016/S0376-8716(02)00217-X 12324177

[ref22] Conesa P. J. Onandia-Hinchado I. Duñabeitia J. A. Moreno M. A. (2022). Basic psychological needs in the classroom: a literature review in elementary and middle school students. Learn. Motiv. 79:101819. doi: 10.1016/j.lmot.2022.101819

[ref23] Costa S. Ntoumanis N. Bartholomew K. J. (2015). Predicting the brighter and darker sides of interpersonal relationships: does psychological need thwarting matter? Motiv. Emot. 39, 11–24. doi: 10.1007/s11031-014-9427-0

[ref24] Credé M. Phillips L. A. (2011). A meta-analytic review of the motivated strategies for learning questionnaire. Learn. Individ. Differ. 21, 337–346. doi: 10.1016/j.lindif.2011.03.002

[ref25] de la Fuente-Arias J. (2017). Theory of self- vs. externally-regulated learning™: fundamentals, evidence, and applicability. Front. Psychol. 8:1675. doi: 10.3389/fpsyg.2017.01675, PMID: 29033872 PMC5627139

[ref26] Deci E. L. Ryan R. M. (2000). The “what” and “why” of goal pursuits: human needs and the self-determination of behaviour. Psychol. Inq. 11, 227–268. doi: 10.1207/S15327965PLI1104_01

[ref27] Deci E. Ryan R. (eds) (2002). Handbook of self-determination research. Rochester, NY: University of Rochester Press.

[ref28] Deci E. L. Ryan R. M. Williams G. C. (1996). Need satisfaction and the self-regulation of learning. Learn. Individ. Differ. 8, 165–183. doi: 10.1016/S1041-6080(96)90013-8

[ref29] Dent A. L. Koenka A. C. (2016). The relation between self-regulated learning and academic achievement across childhood and adolescence: a meta-analysis. Educ. Psychol. Rev. 28, 425–474. doi: 10.1007/s10648-015-9320-8

[ref30] Dignath C. Büttner G. (2008). Components of fostering self-regulated learning among students. A meta-analysis on intervention studies at primary and secondary school level. Metacogn. Learn. 3, 231–264. doi: 10.1007/s11409-008-9029-x

[ref31] Dignath C. Büttner G. Langfeldt H. (2008). How can primary school students learn self-regulated learning strategies most effectively? A meta-analysis on self-regulation training programmes. Educ. Res. Rev. 3, 101–129. doi: 10.1016/j.edurev.2008.02.003

[ref32] Duckworth K. Akerman R. Mac Gregor A. Salter E. Vorhaus J. (2011). Self-regulated learning: a literature review. London, Centre for Research on the wider benefits of learning, Institute of Education.

[ref33] Efklides A. (2011). Interactions of metacognition with motivation and affect in self-regulated learning: the MASRL model. Educ. Psychol. 46, 6–25. doi: 10.1080/00461520.2011.538645

[ref34] Eisenberg N. (2000). Emotion, regulation, and moral development. Annu. Rev. Psychol. 51, 665–697. doi: 10.1146/annurev.psych.51.1.665 10751984

[ref35] Eisenberg N. (2010). “Empathy-related responding: links with self-regulation, moral judgment, and moral behavior” in Prosocial motives, emotions, and behavior: The better angels of our nature. eds. Mikulincer M. Shaver P. R. (Washington: APA), 129–148.

[ref36] Eisenberg N. Spinrad T. L. (2004). Emotion-related regulation: sharpening the definition. Child Dev. 75, 334–339. doi: 10.1111/j.1467-8624.2004.00674.x, PMID: 15056187

[ref37] Eldreth D. Hardin M. G. Pavletic N. Ernst M. (2013). Adolescent transformations of behavioral and neural processes as potential targets for prevention. Prev. Sci. 14, 257–266. doi: 10.1007/s11121-012-0322-1, PMID: 23404658

[ref38] Fraser B. J. (2012). Classroom Environment. London, UK: Routledge.

[ref39] Fredricks J. A. Blumenfeld P. C. Paris A. H. (2004). School engagement: potential of the concept, state of the evidence. Rev. Educ. Res. 74, 59–109. doi: 10.3102/00346543074001059

[ref40] Garcia T. Pintrich P. R. (1994). “Regulating motivation and cognition in the classroom: the role of self-schemas and self-regulatory strategies,” in Self-regulation of learning and performance: Issues and educational applications. eds. Schunk D. H. Zimmerman B. J. (Hillsdale, NJ: Lawrence Erlbaum Associates), 127–153.

[ref41] Garcia T. Pintrich P. R. (1995). Assessing students’ motivation and learning strategies: the motivated strategies for learning questionnaire. Paper presented at the annual meeting of the American Educational Research Association, San Francisco, CA.

[ref42] Gardner T. W. Dishion T. J. Connell A. (2008). Adolescent self-regulation as resilience: resistance to antisocial behavior within the deviant peer context. J. Abnorm. Child Psychol. 36, 273–284. doi: 10.1007/s10802-007-9176-6, PMID: 17899361

[ref43] Garn A. C. Morin A. J. S. Lonsdale C. (2019). Basic psychological need satisfaction toward learning: a longitudinal test of mediation using bifactor exploratory structural equation modeling. J. Educ. Psychol. 111, 354–372. doi: 10.1037/edu000028

[ref44] Gillebaart M. (2018). The ‘operational’ definition of self-control. Front. Psychol. 9:1231. doi: 10.3389/fpsyg.2018.01231, PMID: 30072939 PMC6058080

[ref45] Hadwin A. F. Järvelä S. Miller M. (2011). “Self-regulated, co-regulated, and socially shared regulation of learning,” in Handbook of self-regulation of learning and performance. eds. Zimmerman B. J. Schunk D. H. (New York/London: Routledge/Taylor & Francis Group), 65–84.

[ref46] Hadwin A. Järvelä S. Miller M. (2018). “Self-regulation, co-regulation, and shared regulation in collaborative learning environments,” in Handbook of self-regulation of learning and performance. 2nd Edn. eds. Schunk D. H. Greene J. A. (New York/London: Routledge/Taylor & Francis Group), 83–106.

[ref47] Hampson S. E. Edmonds G. W. Barckley M. Goldberg L. R. Dubanoski J. P. Hillier T. A. (2016). A big five approach to self-regulation: personality traits and health trajectories in the Hawaii longitudinal study of personality and health. Psychol. Health Med. 21, 152–162. doi: 10.1080/13548506.2015.1061676, PMID: 26196294 PMC4718866

[ref48] Hofmann W. Luhmann M. Fisher R. R. Vohs K. D. Baumeister R. F. (2014). Yes, but are they happy? Effects of trait self-control on affective well-being and life satisfaction. J. Pers. 82, 265–277. doi: 10.1111/jopy.12050, PMID: 23750741

[ref49] Housman D. K. Denham S. A. Cabral H. (2018). Building young children’s emotional competence and self- regulation from birth: the begin to … ECSEL approach. Int. J. Emot. Educ. 10, 5–25.

[ref50] Jang H. Kim E. J. Reeve J. (2016). Why students become more engaged or more disengaged during the semester: a self-determination theory dual-process model. Learn. Instruct. 43, 27–38. doi: 10.1016/j.learninstruc.2016.01.002

[ref51] Jansen R. S. van Leeuwen A. Janssen J. Jak S. (2019). Self-regulated learning partially mediates the effect of self-regulated learning interventions on achievement in higher education: a meta-analysis. Educ. Res. Rev. 28:100292. doi: 10.1016/j.edurev.2019.100292

[ref52] Järvelä S. Hadwin A. F. (2013). New frontiers: regulating learning in CSCL. Educ. Psychol. 48, 25–39. doi: 10.1080/00461520.2012.748006

[ref53] Kulik J. A. (2001). Student ratings: validity, utility, and controversy. N. Direct. Inst. Res. 2001, 9–25. doi: 10.1002/ir.1

[ref54] Lakens D. (2013). Calculating and reporting effect sizes to facilitate cumulative science: a practical primer for t-tests and ANOVAs. Front. Psychol. 4:863. doi: 10.3389/fpsyg.2013.00863, PMID: 24324449 PMC3840331

[ref55] Luciana M. (2010). Adolescent brain development: current themes and future directions: introduction to the special issue. Brain Cogn. 72, 1–5. doi: 10.1016/j.bandc.2009.11.002, PMID: 20006416

[ref56] Matos L. Reeve J. Herrera D. Claux M. (2018). Students’ agentic engagement predicts longitudinal increases in perceived autonomy-supportive teaching: the squeaky wheel gets the grease. J. Exp. Educ. 86, 579–596. doi: 10.1080/00220973.2018.1448746

[ref57] Moè A. Consiglio P. Katz I. (2022). Exploring the circumplex model of motivating and demotivating teaching styles: the role of teacher need satisfaction and need frustration. Teach. Teach. Educ. 118:103823. doi: 10.1016/j.tate.2022.103823

[ref58] Moffitt T. E. Arseneault L. Belsky D. Dickson N. Hancox R. J. Harrington H. L.. (2011). A gradient of childhood self-control predicts health, wealth, and public safety. Proc. Natl. Acad. Sci. U. S. A. 108, 2693–2698. doi: 10.1073/pnas.1010076108, PMID: 21262822 PMC3041102

[ref59] Mouratidis A. A. Vansteenkiste M. Michou A. Lens W. (2013). Perceived structure and achievement goals as predictors of students’ self-regulated learning and affect and the mediating role of competence need satisfaction. Learn. Individ. Differ. 23, 179–186. doi: 10.1016/j.lindif.2012.09.001

[ref60] Murray D. W. Kurian J. Soliday Hong S. L. Hong A. Andrade F. C. (2022). Meta-analysis of early adolescent self-regulation interventions: moderation by intervention and outcome type. J. Adolesc. 94, 101–117. doi: 10.1002/jad.12010, PMID: 35353414

[ref61] Murray D. W. Rosanbalm K. Christopoulos C. (2016). Self-regulation and toxic stress report 3: A comprehensive review of self-regulation interventions (OPRE report # 2016–34). Washington, DC: Office of Planning, Research and Evaluation, Administration for Children and Families, U.S. Department of Health and Human Services.

[ref62] Murray D. M. Rosanbalm K. Christopoulos C. Hamoudi A. (2015). Self-regulation and toxic stress report 1: Foundations for understanding self-regulation from an applied perspective (OPRE report # 2015–21). Washington, DC: Office of Planning, Research and Evaluation, Administration for Children and Families, U.S. Department of Health and Human Services.

[ref63] Newman B. M. Newman P. R. (2020). “Self-regulation theories,” in Theories of adolescent development. eds. Newman M. B. Newman P. R. (Amsterdam: Elsevier/Academic Press), 213–243.

[ref64] Núñez J. C. Herrero E. T. Fernández E. Añón F. Manalo E. Rosário P. (2021). Effect of an intervention in self-regulation strategies on academic achievement in elementary school: a study of the mediating effect of self-regulatory activity. Revista de Psicodidáctica 27, 9–20. doi: 10.1016/j.psicoe.2021.09.001

[ref65] Nunnally J. C. (1978). Psychometric theory. New York: McGraw-Hill.

[ref66] Oldehinkel A. J. Hartman C. A. De Winter A. F. Veenstra R. Ormel J. (2004). Temperament profiles associated with internalizing and externalizing problems in preadolescence. Dev. Psychopathol. 16, 421–440. doi: 10.1017/S0954579404044591 15487604

[ref67] Opdenakker M.-C. (2020). “Three decades of educational effectiveness research in Belgium and the Netherlands: key studies, main research topics and findings,” in International perspectives in educational effectiveness research. eds. Hall J. Lindorff A. Sammons P. (Cham: Springer), 231–286.

[ref68] Opdenakker M.-C. (2021). Need-supportive and need-thwarting teacher behavior: their importance to boys’ and girls’ academic engagement and procrastination behavior. Front. Psychol. 12:628064. doi: 10.3389/fpsyg.2021.628064, PMID: 33776849 PMC7988229

[ref69] Opdenakker M.-C. (2022). “Teacher behaviour and student motivational outcomes: critical reflections on the knowledge base and on future research,” in Effective teaching around the world – Theoretical, empirical, methodological and practical insights. eds. Maulana R. Helms-Lorenz M. Klassen R. (Switzerland: Springer)

[ref70] Opdenakker M.-C. Maulana R. den Brok P. (2012). Teacher-student interpersonal relationships and academic motivation within one school year: developmental changes and linkage. Sch. Eff. Sch. Improv. 23, 95–119. doi: 10.1080/09243453.2011.619198

[ref71] Opdenakker M.-C. Van Damme J. (2006a). Differences between secondary schools: a study about school context, group composition, school practice, and school effects with special attention to public and Catholic schools and types of schools. Sch. Eff. Sch. Improv. 17, 87–117. doi: 10.1080/09243450500264457

[ref72] Opdenakker M.-C. Van Damme J. (2006b). Teacher characteristics and teaching styles as effectiveness enhancing factors of classroom practice. Teach. Teach. Educ. 22, 1–21. doi: 10.1016/j.tate.2005.07.008

[ref73] Opdenakker M.-C. Van Damme J. (2007). Do school context, student composition and school leadership affect school practice and outcomes in secondary education? Br. Educ. Res. J. 33, 179–206. doi: 10.1080/01411920701208233

[ref74] Pajares F. (2008). “Motivational role of self-efficacy beliefs in self-regulated learning,” in Motivation and self-regulated learning: Theory, research, and applications. eds. Schunk D. H. Zimmerman B. J. (New York: Erlbaum), 111–139.

[ref75] Pallant J. (2011). SPSS survival manual: A step by step guide to data analysis using SPSS (4th edtn.). Crows Nest, N.S.W.: Allen & Unwin.

[ref76] Panadero E. (2017). A review of self-regulated learning: six models and four directions for research. Front. Psychol. 8, 422. doi: 10.3389/fpsyg.2017.00422, PMID: 28503157 PMC5408091

[ref77] Patall E. A. Steingut R. R. Vasquez A. C. Trimble S. S. Pituch K. A. Freeman J. L. (2018). Daily autonomy supporting or thwarting and students’ motivation and engagement in the high school science classroom. J. Educ. Psychol. 110, 269–288. doi: 10.1037/edu0000214

[ref78] Pintrich P. R. (1999). The role of motivation in promoting and sustaining self-regulated learning. Int. J. Educ. Res. 31, 459–470. doi: 10.1016/S0883-0355(99)00015-4

[ref79] Pintrich P. R. (2000). “The role of goal orientation in self-regulated learning,” in Handbook of self-regulation. eds. Boekaerts M. Pintrich P. R. Zeidner M. (San Diego, CA: Academic Press), 452–502.

[ref80] Pintrich P. R. De Groot E. V. (1990). Motivational and self-regulated learning components of classroom academic performance. J. Educ. Psychol. 82, 33–40. doi: 10.1037/0022-0663.82.1.33

[ref81] Pintrich P. R. Zusho A. (2002). “The development of academic self-regulation: the role of cognitive and motivational factors,” in Development of achievement motivation. eds. Wigfield A. Eccles J. S. (San Diego, CA: Academic Press), 249–284.

[ref82] Rasbash J. Steele F. Browne W. Goldstein H. (2012). A User’s guide to Mlwi N, v2.26. Bristol: Centre for Multilevel Modelling, University of Bristol.

[ref83] Reeve J. (2006). “Extrinsic rewards and inner motivations,” in Handbook of classroom management: Research, practice, and contemporary issues. eds. Weinstein C. Good T. L. (Hillsdale, NJ: Erlbaum), 645–664.

[ref84] Reeve J. (2009). Why teachers adopt a controlling motivating style toward students and how they can become more autonomy supportive. Educ. Psychol. 44, 159–175. doi: 10.1080/00461520903028990

[ref85] Reeve J. Deci E. L. (1996). Elements within the competitive situation that affect intrinsic motivation. Pers. Soc. Psychol. Bull. 22, 24–33. doi: 10.1177/0146167296221003

[ref86] Roelle J. Nowitzki C. Berthold K. (2017). Do cognitive and metacognitive processes set the stage for each other? Learn. Instr. 50, 54–64. doi: 10.1016/j.learninstruc.2016.11.009

[ref87] Roth A. Ogrin S. Schmitz B. (2016). Assessing self-regulated learning in higher education: a systematic literature review of self-report instruments. Educ. Assess. Eval. Account. 28, 225–250. doi: 10.1007/s11092-015-9229-2

[ref88] Ryan R. M. Deci E. L. (2000). Self-determination theory and the facilitation of intrinsic motivation, social development, and well-being. Am. Psychol. 55, 68–78. doi: 10.1037/0003-066x.55.1.68, PMID: 11392867

[ref89] Ryan R. M. Deci E. L. (2020). Intrinsic and extrinsic motivation from a self-determination theory perspective: definitions, theory, practices, and future directions. Contemp. Educ. Psychol. 61:101860. doi: 10.1016/j.cedpsych.2020.101860

[ref90] Ryan A. M. Patrick H. (2001). The classroom social environment and changes in adolescents’ motivation and engagement during middle school. Am. Educ. Res. J. 38, 437–460. doi: 10.3102/00028312038002437

[ref91] Schuitema J. Peetsma T. van der Veen I. (2012). Self-regulated learning and students’ perceptions of innovative and traditional learning environments: a longitudinal study in secondary education. Educ. Stud. 38, 397–413. doi: 10.1080/03055698.2011.643105

[ref92] Schuitema J. Peetsma T. van der Veen I. (2016). Longitudinal relations between perceived autonomy and social support from teachers and students’ self-regulated learning and achievement. Learn. Individ. Differ. 49, 32–45. doi: 10.1016/j.lindif.2016.05.006

[ref93] Schunk D. H. Greene J. A. (2018). Handbook of self-regulation of learning and performance. 2nd edtn. Edn, New York/London: Routledge/Taylor & Francis Group.

[ref94] Sierens E. Vansteenkiste M. Goossens L. Soenens B. Dochy F. (2009). The synergistic relationship of perceived autonomy-support and structure in the prediction of self-regulated learning. Br. J. Educ. Psychol. 79, 57–68. doi: 10.1348/000709908X304398, PMID: 18466671

[ref95] Sijtsma K. (2009). On the use, the misuse, and the very limited usefulness of Cronbach’s alpha. Psychometrika 74, 107–120. doi: 10.1007/s11336-008-9101-0, PMID: 20037639 PMC2792363

[ref96] Skinner E. A. Belmont M. J. (1993). Motivation in the classroom: reciprocal effects of teacher behavior and student engagement across the school year. J. Educ. Psychol. 85, 571–581. doi: 10.1037/0022-0663.85.4.571

[ref97] Skinner E. Furrer C. Marchand G. Kindermann T. (2008). Engagement and disaffection in the classroom: part of a larger motivational dynamic? J. Educ. Psychol. 100, 765–781. doi: 10.1037/a0012840

[ref98] Skinner E. A. Pitzer J. R. (2012). “Developmental dynamics of student engagement, coping, and everyday resilience,” in Handbook of research on student engagement. eds. Christenson S. L. Reschly A. L. Wylie C. (New York, NY: Springer), 21–44.

[ref99] Sparks C. Dimmock J. Lonsdale C. Jackson B. (2016). Modeling indicators and outcomes of students’ perceived teacher relatedness support in high school physical education. Psychol. Sport Exerc. 26, 71–82. doi: 10.1016/j.psychsport.2016.06.004 28787252

[ref100] Steel P. (2007). The nature of procrastination: a meta-analytic and theoretical review of quintessential self-regulatory failure. Psychol. Bull. 133, 65–94. doi: 10.1037/0033-2909.133.1.65, PMID: 17201571

[ref101] Stroet K. Opdenakker M.-C. Minnaert A. (2013). Effects of need supportive teaching on early adolescents’ motivation and engagement: a review of the literature. Educ. Res. Rev. 9, 65–87. doi: 10.1016/j.edurev.2012.11.003

[ref102] Stroet K. Opdenakker M.-C. Minnaert A. (2015). What motivates early adolescents for school? A longitudinal analysis of associations between observed teaching and motivation. Contemp. Educ. Psychol. 42, 129–140. doi: 10.1016/j.cedpsych.2015.06.002

[ref103] Struyven K. Dochy F. Janssens S. Schelfhout W. Gielen S. (2006). On the dynamics of students’ approaches to learning: the effects of the teaching/learning environment. Learn. Instr. 16, 279–294. doi: 10.1016/j.learninstruc.2006.07.001

[ref104] Theobald M. (2021). Self-regulated learning training programs enhance university students’ academic performance, self-regulated learning strategies, and motivation: a meta-analysis. Contemp. Educ. Psychol. 66:101976. doi: 10.1016/j.cedpsych.2021.101976

[ref105] Torrano F. González-Torres M. C. (2004). Self-regulated learning: current and future directions. Electr. J. Res. Educ. Psychol. 2, 1–34.

[ref106] Tuero E. Núñez J. C. Vallejo G. Fernández M. P. Añón F. J. Moreira T.. (2022). Short and long-term effects on academic performance of a school-based training in self-regulation learning: a three-level experimental study. Front. Psychol. 13:889201. doi: 10.3389/fpsyg.2022.889201, PMID: 35645884 PMC9134005

[ref107] Van der Veen I. Peetsma T. T. D. (2009). The development in self-regulated learning behaviour of first-year students in the lowest level of secondary school in the Netherlands. Learn. Individ. Differ. 19, 34–46. doi: 10.1016/j.lindif.2008.03.001

[ref108] Vancouver J. B. (2018). “Self-efficacy’s role in unifying self-regulation theories,” in Advances in motivation science. Vol. 5. ed. Elliot A. J. (London: Elsevier/Academic Press), 203–230.

[ref109] Vandenkerckhove B. Soenens B. Van der Kaap-Deeder J. Brenning K. Luyten P. Vansteenkiste M. (2019). The role of weekly need-based experiences and self-criticism in predicting weekly academic (mal)adjustment. Learn. Individ. Dif. 69, 69–83. doi: 10.1016/j.lindif.2018.11.009

[ref110] Vansteenkiste M. Ryan R. M. (2013). On psychological growth and vulnerability: basic psychological need satisfaction and need frustration as a unifying principle. J. Psychother. Integr. 23, 263–280. doi: 10.1037/a0032359

[ref111] Vansteenkiste M. Ryan R. M. Soenens B. (2020). Psychological need theory: advancements, critical themes, and future directions. Motiv. Emot. 44, 1–31. doi: 10.1007/s11031-019-09818-1

[ref112] Vansteenkiste M. Sierens E. Goossens L. Soenens B. Dochy F. Mouratidis A.. (2012). Identifying configurations of perceived teacher autonomy support and structure: associations with self-regulated learning, motivation and problem behavior. Learn. Instr. 22, 431–439. doi: 10.1016/j.learninstruc.2012.04.002

[ref113] Vansteenkiste M. Soenens B. Sierens E. Luyckx K. Lens W. (2009). Motivational profiles from a self-determination perspective: the quality of motivation matters. J. Educ. Psychol. 101, 671–688. doi: 10.1037/a0015083

[ref114] Vansteenkiste M. Zhou M. Lens W. Soenens B. (2005). Experiences of autonomy and control among Chinese learners: vitalizing or immobilizing? J. Educ. Psychol. 97, 468–483. doi: 10.1037/0022-0663.97.3.468

[ref115] Venitz L. Perels F. (2018). Promoting self-regulated learning of preschoolers through indirect intervention: a two-level approach. Early Child Dev. Care 189, 2057–2070. doi: 10.1080/03004430.2018.1434518

[ref116] Vermote B. Aelterman N. Beyers W. Aper L. Buysschaert F. Vansteenkiste M. (2020). The role of teachers’ motivation and mindsets in predicting a (de) motivating teaching style in higher education: a circumplex approach. Motiv. Emot. 44, 270–294. doi: 10.1007/s11031-020-09827-5

[ref117] Wigfield A. Klauda S. L. Cambria J. (2011). “Influences on the development of academic self-regulatory processes,” in Handbook of self-regulation of learning and performance. eds. Zimmerman B. J. Schunk D. H. (New York/London: Routledge/Taylor & Francis Group), 33–48.

[ref118] Williams G. C. Deci E. L. (1996). Internalization of biopsychosocial values by medical students: a test of self-determination theory. J. Pers. Soc. Psychol. 70, 767–779. doi: 10.1037/0022-3514.70.4.767, PMID: 8636897

[ref119] Winne P. H. (2005). A perspective on state-of-the-art research on self-regulated learning. Instr. Sci. 33, 559–565. doi: 10.1007/s11251-005-1280-9

[ref120] Winne P. H. Hadwin A. F. (2008). “The weave of motivation and self-regulated learning,” in Motivation and self-regulated learning: Theory, research and applications. eds. Schunk D. H. Zimmerman B. J. (New York, NY: Lawrence Erlbaum Associates), 297–314.

[ref121] Winne P. H. Perry N. E. (2000). “Measuring self-regulated learning,” in Handbook of self-regulation. eds. Boekaerts M. Pintrich P. R. Zeidner M. (Orlando, FL: Academic Press), 531–566.

[ref122] Yin H. Lee J. C. Zhang Z. (2009). Examining Hong Kong students’ motivational beliefs, strategy use and their relations with two relational factors in classrooms. Educ. Psychol. 29, 685–700. doi: 10.1080/01443410903218844

[ref123] Zimmerman B. J. (1989). A social cognitive view of self-regulated academic learning. J. Educ. Psychol. 81, 329–339. doi: 10.1037/0022-0663.81.3.329

[ref124] Zimmerman B. J. (1990). Self-regulated learning and academic achievement. Educ. Psychol. 25, 3–17. doi: 10.1207/s15326985ep2501_2

[ref125] Zimmerman B. J. (2000). “Attaining self-regulation: a social cognitive perspective,” in Handbook of self-regulation. eds. Boekaerts M. Pintrich P. R. Zeidner M. (San Diego, CA, US: Academic Press), 13–39.

[ref126] Zimmerman B. J. (2001). “Achieving academic excellence: a self-regulatory perspective,” in The pursuit of excellence through education. ed. Ferrari M. (Mahwah, NJ: Erlbaum), 85–110.

[ref127] Zimmerman B. J. (2008). Investigating self-regulation and motivation: historical background, methodological developments, and future prospects. Am. Educ. Res. J. 45, 166–183. doi: 10.3102/0002831207312909

[ref128] Zimmerman B. J. (2013). From cognitive modeling to self-regulation: a social cognitive career path. Educ. Psychol. 48, 135–147. doi: 10.1080/00461520.2013.794676

[ref129] Zimmerman B. J. (2015). “Self-regulated learning: theories, measures, and outcomes,” in International encyclopedia of the Social & Behavioral Sciences. ed. Wright J. D.. 2nd ed, (Amsterdam: Elsevier) 541–546.

[ref130] Zimmerman B. J. Cleary T. J. (2009). “Motives to self-regulate learning: a social cognitive account,” in Handbook of motivation at school. eds. Wentzel K. R. Wigfield A. (New York: Routledge), 247–264.

